# *SLC25A24* gene methylation and gray matter volume in females with and without conduct disorder: an exploratory epigenetic neuroimaging study

**DOI:** 10.1038/s41398-021-01609-y

**Published:** 2021-09-24

**Authors:** Elizabeth Farrow, Andreas G. Chiocchetti, Jack C. Rogers, Ruth Pauli, Nora M. Raschle, Karen Gonzalez-Madruga, Areti Smaragdi, Anne Martinelli, Gregor Kohls, Christina Stadler, Kerstin Konrad, Graeme Fairchild, Christine M. Freitag, Magdalena Chechlacz, Stephane A. De Brito

**Affiliations:** 1 School of Psychology and Centre for Human Brain Health, University of Birmingham, Birmingham, UK; 2 Department of Child and Adolescent Psychiatry, Psychosomatics, and Psychotherapy, University Hospital Frankfurt, Goethe University Frankfurt, Frankfurt am Main, Germany; 3 School of Psychology and Institute for Mental Health, University of Birmingham, Birmingham, UK; 4 Jacobs Center for Productive Youth Development, University of Zurich, Zurich, Switzerland; 5 Department of Child and Adolescent Psychiatry, King’s College London, London, USA; 6 Child Development Institute, Toronto, ON Canada; 7 RWTH Aachen University, Aachen, Germany; 8 University Psychiatric Clinic Basel, Basel, Switzerland; 9 Department of Psychology, University of Bath, Bath, UK

**Keywords:** Molecular neuroscience, Human behaviour

## Abstract

Conduct disorder (CD), a psychiatric disorder characterized by a repetitive pattern of antisocial behaviors, results from a complex interplay between genetic and environmental factors. The clinical presentation of CD varies both according to the individual’s sex and level of callous-unemotional (CU) traits, but it remains unclear how genetic and environmental factors interact at the molecular level to produce these differences. Emerging evidence in males implicates methylation of genes associated with socio-affective processes. Here, we combined an epigenome-wide association study with structural neuroimaging in 51 females with CD and 59 typically developing (TD) females to examine DNA methylation in relation to CD, CU traits, and gray matter volume (GMV). We demonstrate an inverse pattern of correlation between CU traits and methylation of a chromosome 1 region in CD females (positive) as compared to TD females (negative). The identified region spans exon 1 of the *SLC25A24* gene, central to energy metabolism due to its role in mitochondrial function. Increased *SLC25A24* methylation was also related to lower GMV in multiple brain regions in the overall cohort. These included the superior frontal gyrus, dorsolateral prefrontal cortex, supramarginal gyrus, secondary visual cortex and ventral posterior cingulate cortex, which are regions that have previously been implicated in CD and CU traits. While our findings are preliminary and need to be replicated in larger samples, they provide novel evidence that CU traits in females are associated with methylation levels in a fundamentally different way in CD and TD individuals, which in turn may relate to observable variations in GMV across the brain.

## Introduction

Conduct disorder (CD) is a psychiatric disorder of childhood and adolescence characterized by persistent antisocial behaviors (i.e., violence towards others or animals, destruction of property, theft, and serious rule violations), which significantly impact the individual’s social, academic, or occupational functioning [1]. There is considerable variation in the possible combinations of symptoms that could lead to a CD diagnosis [2]. Therefore, to identify more homogeneous subgroups of youth with CD, several subtyping approaches are included within the Diagnostic and Statistical Manual of Mental Disorders (DSM-5) [1]. One approach focuses on the ‘Limited Prosocial Emotions’ specifier, which indexes callous-unemotional (CU) traits (i.e., reduced empathy, callousness, a lack of guilt, and shallow effect). This specifier designates a particularly impaired subgroup of youths with CD who are at increased risk of developing psychopathy in adulthood [3, 4]. Levels of CU traits show moderate stability from adolescence to adulthood [5] and are also a predictor of more severe antisocial and aggressive behaviors both in adolescence and adulthood [6]. In this context, understanding the etiology of these CU traits in adolescents with CD is an important step towards identifying risk factors for a subgroup of youths with CD who are particularly susceptible to poorer outcomes in adulthood [7].

Research shows that both genetic and environmental risk factors are implicated in the development of conduct problems or CD [8, 9], with around 50% of the variance in CD risk attributable to heritable genetic influences [8]. Crucially, twin studies indicate that youths with CD symptomatology and high versus low levels of CU traits are characterized by different environmental and genetic risk vulnerabilities [4]. Indeed, Viding et al. (2005) demonstrated that antisocial behavior in youths with CD symptomatology and high levels of CU traits is highly heritable (0.76), whereas in youths with CD symptomatology and low levels of CU traits it is moderately heritable (0.64) and more influenced by environmental factors [10]. Along with CU traits, sex is an important factor to consider in youths with CD in relation to genetic vulnerability for this disorder. Indeed, heritability estimates for antisocial behavior in youths with CD are higher in males than females [11]. Furthermore, in males with CD and high levels of CU traits, heritable factors explain a high proportion of the variance in antisocial behavior [10]. Conversely, antisocial behavior in females with conduct problems (CP) and high levels of CU traits was shown to be entirely explained by environmental factors in one study [12]. These data suggest sex differences in the biological mechanisms underlying antisocial behavior in youths with CD depending on their levels of CU traits.

### Gene–environment interplay in CD development

A key question in CD research is how genetic and environmental risk factors interact at the molecular level in relation to CU trait phenotypes [13]. One candidate mechanism is via epigenetic changes in the form of DNA methylation, which involves addition of a methyl group at a specific genomic location [14]. Depending on the pattern, location, and level of methylation within or proximal to the gene’s coding sequence, gene expression may be suppressed or amplified [14]. The genetic variation of an individual is also an important factor to consider in understanding how environmental factors are translated into methylation signatures. Recent research has highlighted that individual differences in heritable factors may influence methylation signatures [15] and thus gene regulation. These genetic variants that can affect DNA methylation are known as methylation quantitative trait loci (mQTLs) and may be further useful markers for genetic influence on gene regulation [16].

Altered regulation of genes expressed in brain tissues and/or implicated in behavior, may explain how methylation levels mechanistically mediate environmental influences, e.g. adverse life experiences to subsequent risk for CD [17] and CU traits [18]. A recent study suggests that exposure to adverse prenatal environmental factors has a large effect on the brain epigenome, and that epigenetic effects associated with brain development are also sex-specific [19].

Epigenetic studies of youths with CD or sub-clinical CP have provided initial evidence that DNA methylation patterns may mediate environmental factors associated with antisocial behavior [20, 21]. In males with CD, methylation of the oxytocin receptor gene (*OXTR*) correlates positively with CU traits [22]. Similarly, in a mixed-sex study, higher methylation of *OXTR* at birth was associated with higher CU traits in adolescence for participants with low levels of anxiety [23]. Alterations in the expression of genes that govern the oxytocin system, as a result of epigenetic modifications, may thus play an important biological role in the development of CD and CU traits [22, 23]. A recent small-scale epigenetic neuroimaging study on males with CD showed that *OXTR* methylation and levels of CU traits interacted to predict frontoparietal hyperactivity and weaker amygdalo-frontoparietal connectivity in males during a face-processing task [24]. This is consistent with previous reports of abnormalities in this circuitry in CD (e.g., [25]) and the fact that *OXTR* is highly expressed in both limbic and cortical brain tissues [26]. Interestingly, a fundamentally opposite association between brain functional connectivity and level of CU traits was observed in CD as compared to TD youths [24].

### Study aims

To expand current knowledge on epigenetics in CD and limited research on females with CD, we adopted an exploratory approach and conducted the first Epigenome-Wide Association Study (EWAS) with salivary DNA data on females with CD and varying levels of CU traits. As previous research in psychiatric disorders has demonstrated differential methylation according to diagnostic status [27] and level of CU traits [22, 28], we first examined the main effects of CD diagnostic status and level of CU traits. Secondly, we [29] and others [24] have demonstrated an inverse association between biomarkers and the level of CU traits in clinical groups as compared to TD populations. Thus, we investigated whether there was a CDxCU traits interaction effect on DNA methylation. The relationship between CU traits and methylation level has been demonstrated in individuals with CD [22, 23] but the nature and direction of this relationship in TD youth is unknown. Finally, to investigate whether these methylation changes co-incidence with altered brain development, we related our methylation data to gray matter volume as measured using voxel-based morphometry (VBM).

## Methods and materials

### Participants

Fifty-one females with CD (mean age = 14.9, SD = 1.7) and 59 TD females (mean age = 14.7, SD = 2.4), recruited across five sites, were included as a subsample of the FemNAT-CD study [30] (see Supplementary Tables S1 for details). This study was conducted according to the legal regulations outlined by the European Union, national legislation, and the Declaration of Helsinki. For each site, written informed consent was obtained from all participants and their parents, in accordance with the site-specific ethical requirements. In addition to standard FemNAT-CD inclusion and exclusion criteria (see Supplementary materials), participants were required to be non-smokers, be medication-free, and have good quality saliva-DNA and structural MRI data. Participants were included in the CD group if they either; (a) met the DSM-5 criteria for a diagnosis of CD; (b) were 9–12 years old, met the criteria for a diagnosis of oppositional defiant disorder (ODD) and also had at least one current symptom of CD; or (c) were aged >12 years, met the criteria for ODD and also had at least 2 current CD symptoms. All TD participants had no diagnosable psychiatric disorders and no history of externalizing disorders (ADHD, ODD). The participants were aged 9–18 years and groups were matched on pubertal development status, performance IQ, ethnicity, and data-collection site (Table 1 and Supplementary Table S1).

**Table 1:** Demographic and clinical characteristics of the participants.

Demographic & Clinical Characteristics	CD (*n* = 51)		TD (*n* = 59)		*P* (*t-*test)	Wilcoxon’s *p*
	*M*	SD	*M*	SD		
*Demographic*						
Age	14.9	1.73	14.7	2.38	0.670	0.961
PDS	3.98	1.05	4.07	0.98	0.651	0.692
SES	−0.540	0.828	0.205	0.902	<0.001	<0.001
Total IQ	94.7	12.2	100.05	10.2	0.013	0.007
Perf. IQ	93.7	14.8	98.83	12.7	0.062	0.091
Verbal IQ	93.6	19.2	101.0	12.9	0.023	0.004
*Clinical*						
ADHD symptoms	0.22	0.42	0.14	0.34	0.24	0.28
GAD symptoms	0.24	0.55	0	0.29	0.008	0.004
MDD symptoms	0.5	0.70	0	0	<0.001	<0.001
ICU total	29.6	11.4	17.6	9.02	<0.001	<0.001
ICU callous	10.2	5.38	4.65	3.80	<0.001	<0.001
ICU uncaring	13.1	5.27	8.37	4.54	<0.001	<0.001
ICU unemotional	6.31	3.59	4.93	2.74	0.030	0.040

*CD* conduct disorder, *TD* typically developing, *PDS* Pubertal Development Scale, *SES* socio-economic status, *IQ* intelligent quotient, *ADHD* attention-deficit/hyperactivity disorder, *GAD* generalized anxiety disorder, *MDD* major depressive disorder, *ICU* inventory of callous-unemotional trait.

### Clinical and psychometric measures

Detailed information about these measures is provided in our previous work [31]. Briefly, trained staff interviewed the participants and their parents (or caregivers) separately using the Schedule for Affective Disorders and Schizophrenia for School-Age Children-Present and Lifetime version (K-SADS-PL [32]) to assess for CD and other DSM-IV-TR psychiatric disorders. Supplementary questions from the K-SADS-PL (e.g. for ODD/ADHD) were completed if key items were endorsed during the initial screening. CU traits were assessed using the parent-version Inventory of Callous-Unemotional Traits (ICU [33]). Total, verbal and performance IQ was assessed using the Wechsler Abbreviated Scale of Intelligence [34] in the UK and the Wechsler Intelligence Scale for Children, Fifth Edition [35] at other sites. Pubertal status was determined using the Pubertal Development Scale (PDS) [36] completed by the participants (if aged >12 years) or by the parents/caregivers (for participants ≤ 12 years).

### Genome-wide methylation data pre-processing

DNA was extracted from saliva within 7 days of collection using the Oragene OG-500 Kit. DNA quality cutoff was a 260/280 ratio above 1.8. DNA was stored at −80 °C immediately. Genome-wide methylation was measured using the Illumina Infinium HumanMethylationEPIC BeadChip Array at Life & Brain GmbH, Bonn, Germany. Pre-processing was performed in R *version 3.6.0* [37]. Raw.idat files were pre-processed with the *minfi* [38] package (*version1.32.0*) following standard parameter settings (*see* Supplementary Methods). We removed failed and noisy probes as suggested [39], and also probes spanning an SNP with an SNP147 data-base annotated MAF > 10%. Finally, cross-reactive probes were eliminated. Between-array normalization was completed using the *preprocessFunnorm()* function [40] included in the *minfi* package, following standard recommendations. This unsupervised method uses control probes to identify unwanted variation. It then extends the idea of quantile normalization to regresses out components of variation captured by these control probes [40]. This has been shown to be an effective method for removing positional effects [41].

We used ANOVA testing in the normalized methylation data to ensure there were no residual batch effects. As an additional check, we also extracted the first principal component of the methylation data and performed pairwise *T*-tests (with Tukey’s correction for multiple testing) across the batches to confirm there were no correlations between the batch IDs and *M* values.

Heat maps and hierarchical clustering plots based on the Euclidean distance of the top 2000 loci selected by variance in methylation were generated to visually check for outliers and batch effects (Supplementary Fig. 1). The methylation *M*-values were calculated based on the log-transformed ratio of methylated to unmethylated signal-intensities for each locus in line with previous research [42] and we ensured these *M* values were normally distributed across the differentially methylated region (Supplementary Fig. 1). Probes were mapped to their genomic region using the human reference genome hg19.

### MRI acquisition

T1-weighted structural scans were collected at five research sites using MRI scanners all operating with 3 T fields (either Siemens or Philips manufactured) and harmonized acquisition sequences (see refs. [29, 31] and Supplementary materials).

### Pre-processing of the neuroimaging data

Consistent with our previous work [29], SPM12 (www.fil.ion.ucl.ac.uk/spm), Computational Anatomy 12 (*CAT-12*: http://dbm.neuro.uni-jena.de/cat/) and template-o-matic (TOM8 [42]) toolboxes were used to pre-process MRI data (see Supplementary materials).

### Genome-wide methylation statistical analysis

To examine the associations between CD diagnostic status, level of CU traits and genome-wide methylation, we employed linear regression modelling: *M*-values for each CpG site was modelled as a function of CD status, CU traits (total ICU score), and the CDxCU traits interaction effect. Corrections for the effects of age and hormonal contraceptive use were included in the model. Socio-economic status (SES) was not included as a covariate in the DNA methylation analysis on statistical and conceptual grounds (see Supplementary materials for further details).

To identify components of extraneous variation due to unmodelled or unknown latent variables, surrogate variable analysis in R (*sva* package, “leek” method selected) was performed and the two factors identified were included in the final model as covariates. The effect sizes and *p*-value of each predictor (CD-case status, CU-trait levels and CDxCU) were calculated using the suggested Bayesian approach as implemented in the minfi *ebayes* function. *P*-values were then submitted to the Bumphunter algorithm [43] to identify differentially methylated regions (DMRs). We specified different coefficients from the linear regression modelling in the arguments of the Bumphunter function to test separately for: (i) the main effect of CD diagnosis, (ii) the main effect of CU score, and (iii) a CD × CU interaction effect on methylation, while controlling for the main effects of the other two factors. QQ plots were generated to confirm appropriate model fits for each EWAS model (see Supplementary Fig. 2). Correction for multiple testing using the false discovery rate (FDR [44]) was done across the individual probes tested as recommended [45].

### VBM analysis

Since we identified a significant DMR associated with the group-by-CU traits interaction effect on methylation level, we employed the GLM framework to explore the association between GMV and average M-value across probes within the respective DMR. No DMR associated with main effects for CD or CU-traits was identified.

Specifically, GMV was analyzed on a voxel-by-voxel basis, via multiple regressions. PDS, SES, total intracranial volume (TIV), scanning site (dummy coded), and total IQ were included as covariates of no interest. Unlike in the epigenetic analysis, we include SES as a covariate here to allow us to investigate the association between methylation and GMV across our full cohort without the potential confounding effects of SES on GMV that are independent of methylation. At a whole-brain level, inferences were made using a statistical threshold of *p* < 0.05 after family-wise error (FWE) correction for multiple comparisons. We also investigated associations between GMV and *M*-value in four regions of interest (ROIs, bilaterally) where the identified gene of interest *SLC25A24* is highly expressed (Genotype-Tissue Expression [46] GTEx project database, see supplementary material Fig. 3), namely the amygdala, hippocampus, basal ganglia and cerebellum (Supplementary Fig. 5). Masks of these regions were defined based on the Talairach Daemon database using the WFU PickAtlas tool in SPM12 [47]. The MarsBAR toolbox was used to extract mean-cluster and peak-voxel GMV values from significant clusters for each participant. All brain imaging coordinates are reported in the standardized Montreal Neurological Institute (MNI) space.

## Results

### Participant characteristics

As per matching on PDS and performance IQ, CD and TD females did not differ in terms of age, puberty, ethnicity, site and performance IQ, but the CD group had lower full-scale IQs than the TD group (Table 1). The number of ADHD symptoms did not differ between groups, but individuals with CD had significantly more symptoms of a generalized anxiety disorder (GAD) and major depressive disorder (MDD) than the TD participants. Females with CD also had higher total ICU and ICU subscale scores (see Table 1 and Supplementary Fig. 4).

### Power calculation

While we acknowledge that our sample size is rather small for a genome-wide approach, power analysis using the online calculation tool epigenetics.essex.ac.uk/shiny/EPICDNAmPowerCalcs confirmed that our analysis with a sample size of *n* = 110 participants conferred each CpG site tested with ~80% power to detect a difference in methylation at the recommended level for the EPIC array (*p* < 6.21e−05). Two other recent studies have similarly adopted a genome-wide approach to investigating DNA methylation in relation to aggressive behaviours in youth, both using a sample size <*n* = 100 [48, 49].

### Identification of differentially methylated regions

**Fig. 1:**
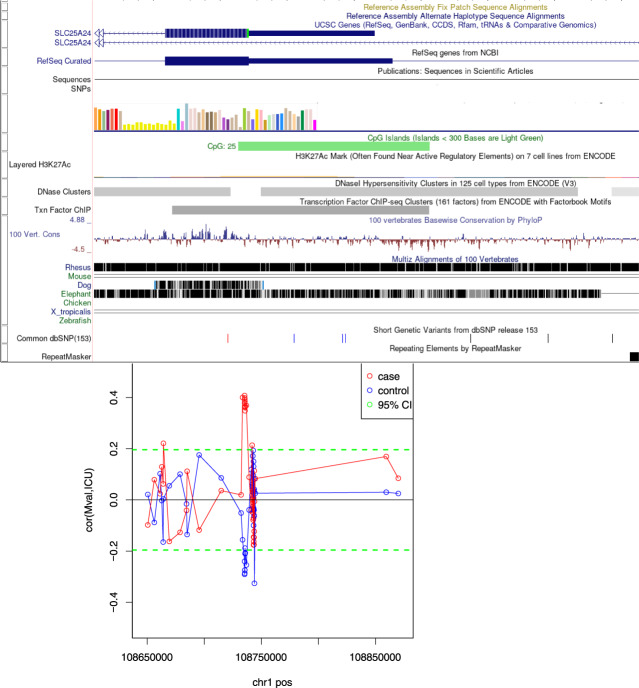
Genomic Location of the Chr1. Differentially Methylated Region. (Top) UCSC Genome Browser Illustration showing stacked annotation tracks beneath the genomic coordinates of the region which showed differential methylation according to the CD × CU traits interaction (from the hg19 human reference gene); (bottom) a scatter plot of this differentially methylated region highlighting the opposite relationship between methylation and level of CU traits in CD cases vs. control participants.

At the single probe level, DNA-methylation was not predicted by case-control status or level of CU traits (at a significance level of *p*_FDR_ < 0.05). However, the CDxCU traits interaction significantly predicted differential methylation at one genomic region on chromosome 1 (hg19 chr1: 108,735,312–108,735,893, FDR = 0.004), spanning eight probes. The interaction was driven by a positive association between CU traits and methylation of the respective probes in females with CD (Pearson *r*_(49)_ = 0.39, *p* = 0.006), but a negative association between CU traits and methylation in TD females (Pearson *r*_(57)_ = −0.27, *p* = 0.042). The slopes of these correlations differed significantly (*Z* = 2.48, *p* = 0.007). The region identified includes exon 1 of the solute carrier *SLC25A24* gene (see Fig. 1).

It is important to note that these methylation findings do not include the methylation values at common SNPs, as these were removed during the pre-processing stage of our analysis, thus our findings should be considered in light of this limitation.

### Association between methylation and gray matter volume

**Fig. 2:**
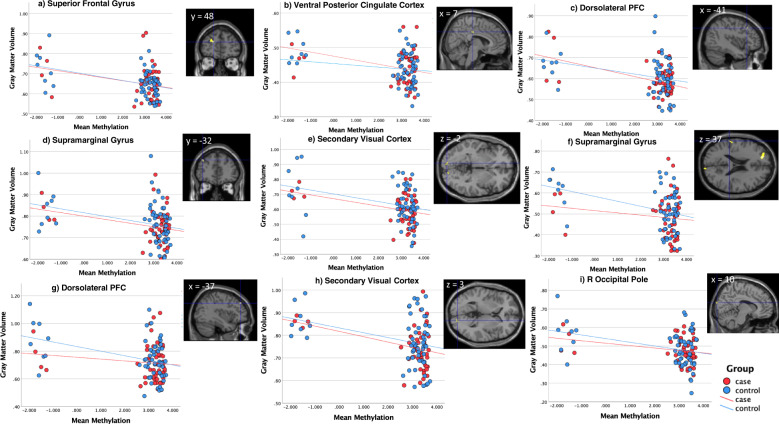
Association between SLC25A24 methylation and gray matter volume. Mean gray matter volume (GMV) values in the cluster significantly associated with methylation for *p* < 0.001, size > 72 voxels were extracted for each participant and then plotted against the average methylation *M*-value across the DMR on chromosome 1 corresponding to exon 1 of gene SLC25A24. CD participants (red) and TD (blue) participants are differentiated by color. In all clusters there is a negative association between GMV and *M* value in both CD and TD groups; the difference between groups in the strength of the correlation is not statistically significant at *p* < 0.05.

We then tested whether the *SLC25A24* methylation levels observed for the interaction effect of CDxCU traits was also associated with GMV in any brain region. After correction for multiple comparisons, no significant (i.e. *p*_FWE_ < 0.05) positive or negative associations between the average *M*-value of the *SLC25A24*-DMR and GMV were detected (in analysis across the whole cohort). However, given the exploratory nature of this study, we report findings at a more liberal significance level of *p* < 0.001 uncorrected with an extent threshold of *k* = 72 voxels empirically determined according to random field theory [50, 51]. At this level we observed a negative association with *SLC25A24* methylation *M*-value for GMV in several clusters within the brain (please see Supplementary Table s2), indicating that higher *SLC25A24* methylation is associated with lower GMV in these regions. We identified these clusters in multiple brain regions including the superior frontal gyrus (SFG), dorsolateral prefrontal cortex (dlPFC), supramarginal gyrus, the secondary visual cortex in the left hemisphere, and the ventral PCC and secondary visual cortex in the right hemisphere. All coordinates are reported in MNI space. Mean cluster GMV values were extracted for each participant and then plotted against the average methylation *M*-value across the DMR on chromosome 1 (i.e. exon 1 of gene *SLC25A24* (see Fig. 2)). Across all regions, in both CD and TD groups, there was a negative association between GMV and the mean exon 1 *SLC25A24* *M*-value.

### ROI analysis

No significant positive or negative association between *SLC25A24* methylation and GMV could be detected in the amygdala, hippocampus, basal ganglia or cerebellum ROIs. (Please see Supplementary Fig. 5 for 3D visualization of the four brain regions tested as ROIs.)

### Post-hoc testing of *OXTR* methylation

We did not observe a significant association between CU traits and methylation at any of the 12 CpG sites on the *OXTR* gene for which we had DNA methylation data. Even when the significance threshold was reduced to a nominal level of *p* < 0.001, uncorrected, the main effect of CU traits was not significant for any of the individual sites, or for this region as a whole.

## Discussion

To our knowledge, this is the first EWAS and epigenetic neuroimaging study in females with CD. First, we examined the main effects of CD group status, level of CU traits and their interaction on saliva-based DNA methylation. Our analyses revealed that in CD and TD females there is a fundamentally opposite pattern of association between CU traits and methylation at a chromosome 1 genomic region, spanning exon 1 of the *SLC25A24* gene. Second, we related the identified DMR to GMV, both in multiple brain regions implicated in CD and CU traits and in a whole-brain exploratory analysis. GMV in regions including the SFG, dlPFC and supramarginal gyrus was negatively correlated with methylation levels, however, these neuroimaging findings did not reach the minimum threshold for significance.

### Genome-wide methylation

We found a significant CD × CU traits interaction effect on methylation level in exon 1 of the *SLC25A24* gene, whereby methylation level was positively correlated with CU traits in CD participants, but negatively correlated with CU traits in TD controls. Elevated methylation at the first exon and promoter regions of genes has been demonstrated to decrease the expression of the respective gene [52, 53]. Thus, our results indicate that in adolescent females with CD, higher levels of CU traits are associated with reduced *SLC25A24* gene expression, whereas in TD females, CU traits are positively associated with gene expression.

*SLC25A24*, a member of a solute-carrier gene family [54], is involved in adenosine triphosphate (ATP)-mediated Calcium buffering at the mitochondrial matrix and is potentially involved in protecting cells against oxidative stress-induced cell death. In mitochondria, ATP production is associated with the production of free oxidative radicals. These cellular redox scavengers, as well as nutrition-derived antioxidants, are crucial to neutralize these free radicals [55]. As the brain accounts for 25% of the body’s total energy expenditure [56], impaired mitochondrial function, as suggested by a reduced expression of *SLC25A24*, may lead to higher rates of cell death due to oxidative stress [57] and thus leave neuronal cells especially vulnerable to oxidative damage [58]. Increased cell death, due to an impaired redox-scavenger system in the brain’s mitochondria, may also, at least partially, explain the association we observed with GMV. Furthermore, unbalanced energy provision and reduced Calcium homeostasis in neurons may result in impaired functioning and ultimately lead to neurodegeneration [57]. Accordingly, mitochondrial dysfunction has been suggested to be associated with several neurodevelopmental disorders, including autism spectrum disorder (ASD) [59, 60] and ADHD [61]. Reduced expression of the *SLC25A24* gene has been reported in the thalamus and motor cortex of patients with ASD and hypothesized to be associated with the impairments in sensory processing and response inhibition observed in this population [62].

As discussed, deficient mitochondrial functioning is a possible consequence of increased methylation and the resulting decreased expression of the *SLC25A24* gene. Given that mitochondria work alongside the mitochondrial-bound monoamine oxidase A (MAO-A) enzyme to break down catecholaminergic neurotransmitters [63], altered functioning of either component in the degradation process may contribute to abnormally high or low levels of neurotransmitters in the brain [64]. Importantly, atypical levels of neurotransmitters have previously been associated with both CD [13] and CU traits [28]. Both elevated SLC25A24 methylation and variants of the MAO-A enzyme may contribute to disrupted catecholamine catabolism. This is reported to be the biological means by which variation of the *MAOA* gene contributes to the affective (e.g., emotion dysregulation) and behavioral (e.g., reactive aggression) features of females with CD [65]. Thus, *SLC25A24* gene hypermethylation may also result in behavioral patterns associated with atypical levels of neurotransmitters in the brain in a similar way to that reported for variants of the MAO-A enzyme, which have previously been linked to aggressive/violent behaviors in both animals [66] and humans [67].

### Environmental risk factors and SLC25A24 methylation

Childhood maltreatment, a key factor known to influence DNA methylation [68], has been shown to interact with MAOA variants to predict aggression in both sexes [69]. In females, the high activity allele has been shown to confer a risk for aggressive behavior following childhood maltreatment [69], but see ref. [70]. Future studies should further investigate the relationship between childhood maltreatment and methylation to determine whether experiences of child maltreatment alter DNA methylation levels and thereby increase the risk for aggressive behaviors.

More generally, mitochondrial dysfunction has been linked to exposure to environmental stressors [71]. Mitochondria are key components of the human body’s stress response system, providing intra-cellular energy and synthesizing stress hormones and neurotransmitters central to stress responding [72]. Experimental manipulation of mitochondrial function has been shown to influence physiological and behavioral responses to psychological stress [72]. Crucially, there is evidence that epigenetic markers of stress exposure are mitochondrially regulated [72]. Thus, reduced expression in genes governing mitochondrial function, such as *SLC25A24*, may arbitrate how environmental factors result in epigenetic modifications [73].

Individuals with CD are more likely to have experienced ‘stressful’ early life environments and thus to have elevated stress biomarkers associated with psychiatric symptoms [74]. CU traits may be another factor that moderates the association between environmental risk factors and the individual’s biological stress response [75]. Consequently, the combination of CD diagnostic status and level of CU traits may influence epigenetic markers associated with stress exposure. Altered methylation across genes in the energy metabolism system may represent an adaptive response to these variations. Thus, rather than being a unique marker of one stressor, we postulate that *SLC25A24* gene methylation may reflect the cumulative effect of exposure to multiple early-life environmental factors triggering the biological stress response system.

### Epigenetic neuroimaging data

Our neuroimaging analysis revealed trend-level negative associations between *SLC25A24* methylation values and GMV in several brain regions, namely, the SFG, dlPFC, supramarginal gyrus and secondary visual cortex in the left hemisphere, and the ventral posterior cingulate cortex (PCC) and secondary visual cortex in the right hemisphere.

These results may suggest that higher levels of SLC25A24 gene methylation is linked to a reduction in GMV in these regions. This finding would be consistent with the theory that increased methylation has a silencing effect on the gene, leading to impaired mitochondrial function (and thus a reduced capacity for energy production and growth) during brain development. Many of the regions where reduced GMV was observed, such as the SFG, dlPFC, the supramarginal gyrus and the ventral PCC, are involved in higher cognitive functions, such as working memory [76], as well as socio-cognitive processes such as affective empathy, which have been shown to be impaired in CD [13, 77]. For example, in a recent meta-analysis of 13 VBM studies, we found that youths with CP had significantly reduced GMV in the left medial SFG [78]. Atypical cortical thickness and functional connectivity have also been reported in adults with psychopathy in several brain regions across the frontal cortices [79] and deficits in cortical folding in these regions are also reported in youths with CD [80].

In youths with CD, greater levels of methylation were observed in association with higher CU traits and greater levels of methylation were also related to reductions in GMV at trend-level. In TD youths, we see the inverse pattern (with individuals with higher CU traits having higher GMV in the observed brain regions). We speculate that in individuals with CD and high CU traits this increased methylation and the associated higher levels of oxidative stress during energy production contributes to a higher rate of neuronal death during neuronal pruning, and subsequently leads to a reduction in GMV in the observed brain regions in this group. However, currently, the underlying factors contributing to this mechanism are unknown, and further research with more highly powered studies is needed to determine whether the suggestive negative relationship between GMV and methylation we observed here holds true in larger samples.

### Post-hoc testing of *OXTR* methylation

The fact that other studies have found an association between CU traits and methylation of the *OXTR* gene (e.g. refs. [22, 23]), but we did not can be explained by a number of factors. For example, this may be related to methodological differences between our study and previous studies, such as the use of different measures of CU traits (i.e., ICU here, but others [24] have used the Youth Psychopathic Traits Inventory (YPI [81]) or other different investigative approaches, i.e. candidate gene vs. epigenome-wide studies. Additionally, we focused on females only, which contrasts with previous studies that have relied on male-only or mixed-sex samples.

### Strengths and limitations

As the first study integrating epigenetic and neuroimaging data from females with CD, this work is an important contribution to our understanding of the biological factors implicated in CD and CU traits in females. Using multi-site data allowed for a larger sample size than would have been possible at a single site, as CD females are difficult to recruit. Furthermore, as data were collected as part of the FemNAT-CD project, the sample is well-characterized, with all participants undergoing thorough assessment for psychiatric disorders and symptoms using a reliable measure based on DSM-IV-TR criteria. Finally, the two groups did not differ on PDS, performance IQ, ADHD symptoms, site and ethnicity, minimizing the potential confounding effects of these factors.

Nevertheless, this study has limitations. First, the sample size is relatively small. As mentioned above, power analysis confirmed our analysis with a sample size of *n* = 110 participants conferred each CpG site tested with ~80% power to detect a difference in methylation at the recommended level for the EPIC array (*p* < 6.21e−05). This power allows us to detect moderate-to-large effects, however smaller effects (*f* < 0.35) on genome-wide methylation levels or GMV were not detectable with this study design. Also, we only had data on childhood maltreatment for a small subset of participants (*n* = 31), so we were unable to include this information in our analysis. Second, while several previous studies report concordance of DNA methylation across saliva and brain tissues (e.g. ref. [82]), tissue-specific epigenetic modifications have also been reported [83]. Thus, it is possible that the differential methylation in salivary DNA demonstrated in this study does not accurately reflect brain-level methylation and might thus be specific to buccal cells only. We also did not correct for cell composition in our salivary DNA samples. Third, as the methylation findings we report do not include the methylation at common SNPs, we do not yet know whether the methylation differences we observe are themselves genetically influenced. Finally, due to funding limitations, we chose to focus solely on investigating genome-wide methylation in females. We felt this would maximise the novelty of our work and add to the knowledge base in this particularly under-researched group. However, as we only included female participants our findings may not apply to males with CD, as research indicates sex-specific influences of environmental and genetic factors on CD and CU traits [10, 12]. Thus similar studies in males and mixed-sex samples will be an important area of future research to investigate whether these mechanisms are sex-specific.

## Conclusions

Methylation of the *SLC25A24* gene was significantly associated with CU traits in both females with CD and TD females but in a fundamentally opposing pattern. Given its essential role in energy metabolism, *SLC25A24* is a key component of the biological stress response system. We postulate that the combination of the individual’s level of CU traits and the number of stressful early life experiences may epigenetically modify the *SLC25A24* gene thus influencing its functionality. Furthermore, we detected negative trends between *SLC25A24* methylation values and GMV in several brain regions, many of which have also been implicated in CD and CU traits. While our findings are preliminary and need to be replicated in larger samples, they provide novel evidence that CU traits in females are associated with methylation levels in a fundamentally different way in CD and TD groups, which in turn relates to observable variations in GMV in the brain.

## Supplementary information

Supplementary Materials
