# Correction: *SLC25A24* gene methylation and gray matter volume in females with and without conduct disorder: an exploratory epigenetic neuroimaging study

**DOI:** 10.1038/s41398-021-01643-w

**Published:** 2021-10-29

**Authors:** Elizabeth Farrow, Andreas G. Chiocchetti, Jack C. Rogers, Ruth Pauli, Nora M. Raschle, Karen Gonzalez-Madruga, Areti Smaragdi, Anne Martinelli, Gregor Kohls, Christina Stadler, Kerstin Konrad, Graeme Fairchild, Christine M. Freitag, Magdalena Chechlacz, Stephane A. De Brito

**Affiliations:** 1 School of Psychology and Centre for Human Brain Health, University of Birmingham, Birmingham, UK; 2 Department of Child and Adolescent Psychiatry, Psychosomatics, and Psychotherapy, University Hospital Frankfurt, Goethe University Frankfurt, Frankfurt am Main, Germany; 3 School of Psychology and Institute for Mental Health, University of Birmingham, Birmingham, UK; 4 Jacobs Center for Productive Youth Development, University of Zurich, Zurich, Switzerland; 5 Department of Child and Adolescent Psychiatry, King’s College London, London, UK; 6 Child Development Institute, Toronto, ON Canada; 7 RWTH Aachen University, Aachen, Germany; 8 University Psychiatric Clinic Basel, Basel, Switzerland; 9 Department of Psychology, University of Bath, Bath, UK

**Keywords:** Molecular neuroscience, Human behaviour

Correction to: *Translational Psychiatry* 10.1038/s41398-021-01609-y, published online 24 September 2021

Since the publication of the article the authors have noticed mistakes in the text, figures, tables and supplementary materials. The authors apologize for these errors, which have now been corrected in the original article. Please note that these changes do not affect the results of the paper or their interpretation.

