# Supplementary material for: SLC25A24 gene methylation and gray matter volume in females with and without conduct disorder: an exploratory epigenetic neuroimaging study
Source: Transl Psychiatry. 2021 Sep 24;11:492. doi: 10.1038/s41398-021-01609-y (PMC8463588; doi:10.1038/s41398-021-01609-y)
Supplement: Supplementary file 1 — Supplementary Materials [file 41398_2021_1609_MOESM1_ESM.docx]

# SLC25A24 Gene Methylation and Gray Matter in Females with and without Conduct Disorder: An Exploratory Epigenetic Neuroimaging Study (Supplement)

Farrow E.^1*^, Chiocchetti, A. G^2^., Rogers, J. C^3^., Pauli, R^1^., Raschle N^4^., Gonzalez-Madruga, K.^5^, Smaragdi, A.^6^, Martinelli, A.^2^ Kohls, G.^7^, Stadler, C^8^., Konrad, K^7^ ,. Fairchild, G.^9^, Freitag, C. M.^2^. Chechlacz, M^1^, De Brito, S. A.^1*^

**Supplementary Methods**

## 1.1 Assessment instruments

**K-SADS-PL.** Current and history of psychiatric disorders (affective, psychotic, anxiety, behavioral, substance abuse, and other psychiatric disorders) were assessed by separate semi-structured diagnostic interviews using The Schedule for Affective Disorders and Schizophrenia for school-age children - Present and Lifetime version (K-SADS-PL)^1^ with participants and their parents and /or caretaker(s). All interviews were conducted by trained masters- and doctoral-level staff. Interrater reliability (Cohen’s κ = .91) and rater agreement (95%) of current CD symptoms were high. Also, for current and past ADHD, oppositional defiant-, depressive and posttraumatic stress disorders, interrater reliabilities (Cohen’s κ = .50 - .95) and rater agreements (92 - 95%) were moderate to high.

**IQ.** IQ was assessed with the Wechsler Abbreviated Scale of Intelligence^2^ for UK sites and the Wechsler Intelligence Scale for Children, Fifth Edition^3^ for all the other sites.

**PDS.** Pubertal status was assessed via self-report using the Pubertal Development Scale^4^ asking about pubertal growth (e.g. changes in body hair, voice or breast development) with four response options (not yet started, barely started, definitely started, seems complete) resulting in a 5-level categorical scale (0 = pre-pubertal, 1 = early-pubertal, 2 = mid-pubertal, 3 = late-pubertal, 4 = post-pubertal).

**Semi-structured psychosocial and medical history.** Psychosocial and medical risk factors were assessed using the Medical History, a semi-structured clinical interview conducted by trained masters- and doctoral-level staff designed to assess current and past environmental influences and exposure to risk factors of children and adolescents. Parents and/or caregivers were interviewed regarding; 1. Pregnancy and birth history, 2. Specific risk factors during the early development, 3. Developmental milestones, 4. Nursery and kindergarten, 5. School career, 6. Chronic medical problems, 7. Parental education status, 8. Information about the family, such as single parenting, and 9. Psychiatric disorders in family.

**Socio-economic status.** Socio-economic status (SES) was assessed based on parental income, education and occupation. Assessments were based on the International Standard Classification of Occupations^5^ and the International Classification of Education (ISCED^6^). Human rater and computer-based ratings were combined into a factor score using Principal Component Analysis (PCA). A clear one-dimensional structure underlying the different measures could be corroborated using Confirmatory Factor Analysis (CFI=.995; RMSEA=0.035). Reliability (internal consistency) of the composite SES score was acceptable (Cronbach’s Alpha =.74). The validity of the SES score could be verified by considering significant correlations with external criteria (e.g. cognitive abilities [r=.33, p<.01], community violence [r=-.28, p<.01]). Due to potential economic variation on the country level, SES was centered and scaled within each country, in order to obtain an indicator of relative socioeconomic position.

## 1.2 Group Matching Procedure

The Match^7^ algorithm was used to create two equal-sized groups of CD and TD participants for whom good quality DNA saliva samples and MRI data was available. The two data files inputted to the algorithm each contained a candidate list of all participants in that group with useable data (i.e. List 1 contained ID numbers of all possible CD participants we could include in our study, list two contained ID numbers of all possible TD participants). Each file also contained the dimensions which we wanted to match the groups on. All variables we had data measures for which had previously been reported to influence neuroimaging or genetic data values were included. These were; Data-collection site, Ethnicity, IQ (including Verbal IQ and Performance IQ subscales), PDS and use of hormonal contraceptives. Match was configured to identify the best-matching 61 items from each file. Items were matched in a pairwise system so that each component of output list 1 had a direct analogue in list 2. Summary statistics for each group were computed to ensure that Match had successfully matched all relevant dimensions, this confirmed that there were statistically no significant differences between the groups in Data-collection site, Ethnicity, Performance IQ, PDS or hormonal contraceptives use.

We obtained DNA methylation data for 122 participants however for the final analysis this number reduced to 110 participants as technical outliers were removed during the epigenetic pre-processing stages. As some participants were excluded, group differences emerged in age and prevalence of hormonal contraceptive use. These variables were corrected for in the final analysis (see section ***DNA Methylation Analysis*).**

## 1.3 MRI data collection procedure and analysis

**MRI acquisition.** MRI data was collected at one of five European sites. Prior to data collection all sites underwent screening testing with phantom and healthy volunteers: this allowed scanning parameters to be adjusted according to site and so ensured that experimental protocols were equivalent, thus confirming images could be used for multi-site analyses. Acquisition of structural MRI data across different sites was coordinated by adherence to a standard operating procedure determined by the FEMNAT-CD project’s steering committee. All structural MRI (sMRI) data was collected using MRI scanners operating with 3T fields (either Siemens or Philips manufactured). Structural T1-weighted images were quality checked in-session and where necessary scanning was repeated to improve image quality.

**1.3 Pre-processing of the neuroimaging data**

Consistent with our previous work^8^, the Computational Anatomy 12 (*CAT-12*) and template-o-matic (*TOM8^9^*) toolboxes were used for the pre-processing of the MRI data. Briefly, customized Tissue Probability Maps (TPMs) were created using the matched pairs approach implemented in TOM8, with participants’ ages as the defining input parameters.

The T1-weighted scans were then segmented in CAT12 with reference to the TPMs, to give separate affine registered gray and white matter segments. Next, these segmented gray matter and white matter images were used to generate a Diffeomorphic Anatomical Registration Through Exponentiated Lie Algebra (*DARTEL^10^*) template, which was normalized to MNI space. Finally, images were smoothed using a 6mm x 6mm x 6mm full-width at half-maximum Gaussian kernel, allowing for an improved signal-to noise ratio in our data and ensuring the General Linear Model (GLM) assumptions were valid. All T1-scans included in the final analysis had a CAT12 quality rating of B- or higher.

**1.4 VBM Analysis using a linear regression model.**

Employing the framework of the GLM, we explored the association between structural brain measures and the average M-value across the region identified in the epigenetic analysis as being differentially methylated by the group-by-CU traits interaction effect. Specifically, Gray Matter Volume (GMV) or White Matter Volume (WMV) were analyzed on a voxel-by-voxel basis, via multiple regressions. PDS, SES, total intra-cranial volume (TIV), scanning site (dummy coded), and total IQ were included as co-variates of no interest. At whole-brain level, inferences were made using a statistical threshold of p < .05 after Family Wise Error (FWE) correction for multiple comparisons. We also investigated associations between GMV and M-value in four regions of interest (ROIs) of the brain where the SLC25A24 gene has been reported to be most highly expressed (Genotype-Tissue Expression (GTEx) project database^11^) Based on the GTEx database, the ROIs tested were the bilateral amygdala, hippocampus, basal ganglia and cerebellum. Masks of these regions were defined based on the Talairach Daemon database using the WFU_PickAtlas tool in SPM12^12^ and a 3D visualization of these regions is shown in supplementary figure 5. For each participant, the MarsBAR toolbox in SPM was used to extract the mean GMV values from each cluster.

## Epigenetic data Pre-processing and DNA methylation analysis

**Genome-wide Methylation Epigenetic data pre-processing.** DNA was extracted from saliva using the Oragene OG-500 Kit, following manufactures protocol. DNA QC included a 260/280 ratio >1.8 and a delay between sample collection and purification of less than 7 days. Samples were frozen to -80°C immediately after purification.

The Illumina Epic Array chip provides information for over 850,000 methylation sites, however after we had completed QC procedures, as detailed in our methods and below in the supplementary methods, the number of sites tested was reduced to ~95% of this number (816,720 CpG sites).

DNA methylation analysis was performed using the statistical programming software R *version 3.6.0^13^* and Bioconductor^14^. Raw idat files were pre-processed implementing the pipeline provided by the *minfi^15^* package (version1.32.0). Removal of failed and noisy probes was done as previously suggested^16^ using the *detectionP()* function in *minfi*, with standard parameter settings. As recommended in the minfi tutorial document^17^ we also chose to remove all probes which contained SNPs (with a MAF > 10%) within the probe body or nucleotide extension, as genotype information was not available for our participants. SNP exclusion was done using standard parameters in the *dropLociWithSnps()* function, which identifies loci of SNPs according to their minor allele fraction (cut-off value of > 10% frequency within the general population specified). Finally, cross-reactive probes were eliminated in line with standard recommendations (e.g.^18^) using the *dropXreactiveLoci()* function with default settings.

Between-array normalization was completed using the *preprocessFunnorm()* function^17^ included in the minfi package with the standard parameters. This algorithm corrects for unwanted variation by regressing out abnormalities due to control probes on the chip. Cleaned data plots were also generated and visually checked to verify that no group or batch effects we present in the data and to identify any technical outliers.

**DNA Methylation Analysis.** The ratios of methylated to unmethylated signal intensities were computed into β values for each locus and these values underwent logit transformation to extract the M-values. The M-value method has been demonstrated to more accurately identify detection rates and true positive rates for methylated genomic loci^19^ than when raw β scores are used. Therefore, the M-value is more statistically suitable for analysis of differential methylation and is thus the only value used in the Epigenetic Neuroimaging stage of this study. Finally, the *AnnotationHub* package in Bioconductor was used for mapping of probes to their genomic region using the human reference genome hg19.

To examine the associations between CD case status, levels of CU traits and genome-wide methylation, we employed linear regression modelling. M values for each CpG site were modelled as a function of CD diagnostic status, total ICU score and the interaction effect of these two factors. To identify the principle components of extraneous variation, surrogate variable analysis in R (*sva* package, “leek” method selected) was performed and the two factors identified were included in the final model as covariates.

$$M \sim\left( Group*ICU total score \right) + Age+Ccept+sv1+sv2$$

SES was not included in the DNA methylation analysis on statistical and conceptual grounds. From a statistical point of view, when using a group design where participants are not randomly allocated to the groups (here CD vs TD), one cannot control or covary out the influence of a third variable (here SES) that might relate to group status. There is indeed agreement in the field of statistics that this approach renders interpretation of results problematic^20^. From a conceptual point of view, nuisance variables such as SES may be critical elements in a complex causal chain contributing to CD. Indeed, we know that CD is associated with lower SES. Evidence also shows that low SES is associated with increased stress in children^21^, which in turn may increase the risk of CD in predisposed persons. In this case, SES is part of the complex causal chain leading to CD. By controlling for this variable, we would have removed one or more of the factors contributing to CD and dampen/wiped out any potential influence of CD on our results.

The final model was tested using the minifi *ebayes* function. This function identified effect sizes of the different predictors of interest (i.e. the main effect of CU and CD as well as the interaction effect thereof) which were then used in the *Bumphunter* algorithm^22^. This algorithm was used to search for differentially methylated regions (DMRs). Bumphunter compares methylation levels across multiple ‘candidate regions’ across the genome to identify where methylation level differs according to the co-efficient(s) of interest, as specified in the inputted design model. We employed this algorithm with our linear regression model design, to identify DMRs for;

(i)Main effect of group,

(ii)Main effect of ICU score, and

(iii)Interaction effect of ICU score and group status.

# Supplementary Tables

**Table S1**: Participant Numbers from each data collection site in the FemNAT-CD study

| **Data Collection Site** | **Number of Participants** | |
| --- | --- | --- |
|  | ***CD*** | ***TD*** |
| 1 | 4 | *3* |
| 2 | 4 | *6* |
| 4 | 12 | 6 |
| 5 | 12 | 17 |
| 7 | 19 | 27 |

**Notes:** Site 1= Universitätsklinikum Aachen, Site 2= Johann Wolfgang Goethe Universität Frankfurt am Main, Site 4= University of Southampton, Site 5= Universität Basel, Site 7 = The University of Birmingham.
Chi-squared testing confirmed no significant association between group and data collection site *X*^2^ (2, *N* = 110) = 4.24, *p* = .375

**Table S2**

| **Covariates** | **Brain region** | **BA** | **L/R** | **Peak Voxel** | **k** | **Z** | **Uncorrected P-value** |
| --- | --- | --- | --- | --- | --- | --- | --- |
|  |  |  |  |  |  |  |  |
| **PDS, SES** | SFG | 10 | L | -17 48 20 | 528 | 4.57 | <.001 |
|  | dlPFC | 9 | L | -37 42 38 | 108 | 3.99 | <.001 |
|  | Supramarginal Gyrus | 40 | L | -48 -32 34 | 290 | 3.74 | <.001 |
|  | Ventral PCC | 23 | R | 7 -24 37 | 235 | 3.57 | <.001 |
|  | Secondary Visual Cortex | 18 | L | -11 -92 -2 | 177 | 3.57 | <.001 |
|  | Secondary Visual Cortex | 18 | R | 17 -87 3 | 140 | 3.54 | <.001 |
|  | dlPFC | 9 | L | -41 23 39 | 84 | 3.37 | <.001 |
|  | Supramarginal Gyrus | 40 | L | -61 -35 21 | 97 | 3.36 | <.001 |
|  | Secondary Visual Cortex | 18 | R | 10 -96 20 | 79 | 3.33 | <.001 |
| **PDS, SES, GAD** | SFG | 10 | L | -17 48 20 | 490 | 4.51 | <.001 |
|  | dlPFC | 9 | L | -37 42 38 | 97 | 3.93 | <.001 |
|  | Angular Gyrus | 39 | L | -43 -46 25 | 73 | 3.90 | <.001 |
|  | Secondary Visual Cortex | 18 | L | -11 -92 -1 | 230 | 3.66 | <.001 |
|  | Supramarginal Gyrus | 40 | L | -48 -32 34 | 101 | 3.65 | <.001 |
|  | Supramarginal Gyrus | 40 | L | -58 -27 38 | 174 | 3.60 | <.001 |
|  | Primary Visual Cortex | 17 | R | 17 -87 3 | 150 | 3.56 | <.001 |
|  | Dorsal PFC | 31 | R | 6 -24 38 | 266 | 3.54 | <.001 |
|  | Supramarginal Gyrus | 40 | L | -57 -28 21 | 214 | 3.53 | <.001 |
|  | Pre-Motor region | 6 | R | 50 -1 53 | 73 | 3.47 | <.001 |
|  | dlPFC | 9 | L | -41 23 39 | 160 | 3.45 | <.001 |
| **PDS, SES, MDD** | SFG | 10 | L | -17 48 20 | 500 | 4.49 | <.001 |
|  | dlPFC | 9 | L | -37 42 38 | 111 | 3.97 | <.001 |
|  | Primary Visual Cortex | 17 | R | 17 -87 3 | 162 | 3.66 | <.001 |
|  | Supramarginal Gyrus | 40 | L | -48 -32 34 | 242 | 3.66 | <.001 |
|  | Secondary Visual Cortex |  | L | -12 -91 -2 | 197 | 3.60 | <.001 |
|  | Ventral PCC | 8 | R | 7 -24 37 | 235 | 3.53 | <.001 |
|  | dlPFC | 9 | L | -41 23 39 | 131 | 3.41 | <.001 |
|  | Supramarginal Gyrus | 40 | L | -60 -34 21 | 152 | 3.41 | <.001 |

*Notes: PDS = Pubertal Development Status, GAD = Generalized Anxiety Disorder, MDD = Major Depressive Disorder, SFG = Superior Frontal Gyrus, dlPFC =Dorso-lateral Prefrontal Cortex, PCC = Posterior Cingulate Cortex*

# Supplementary Figures

**1.a. Complex Heatmap of Genome-Wide Methylation Data**


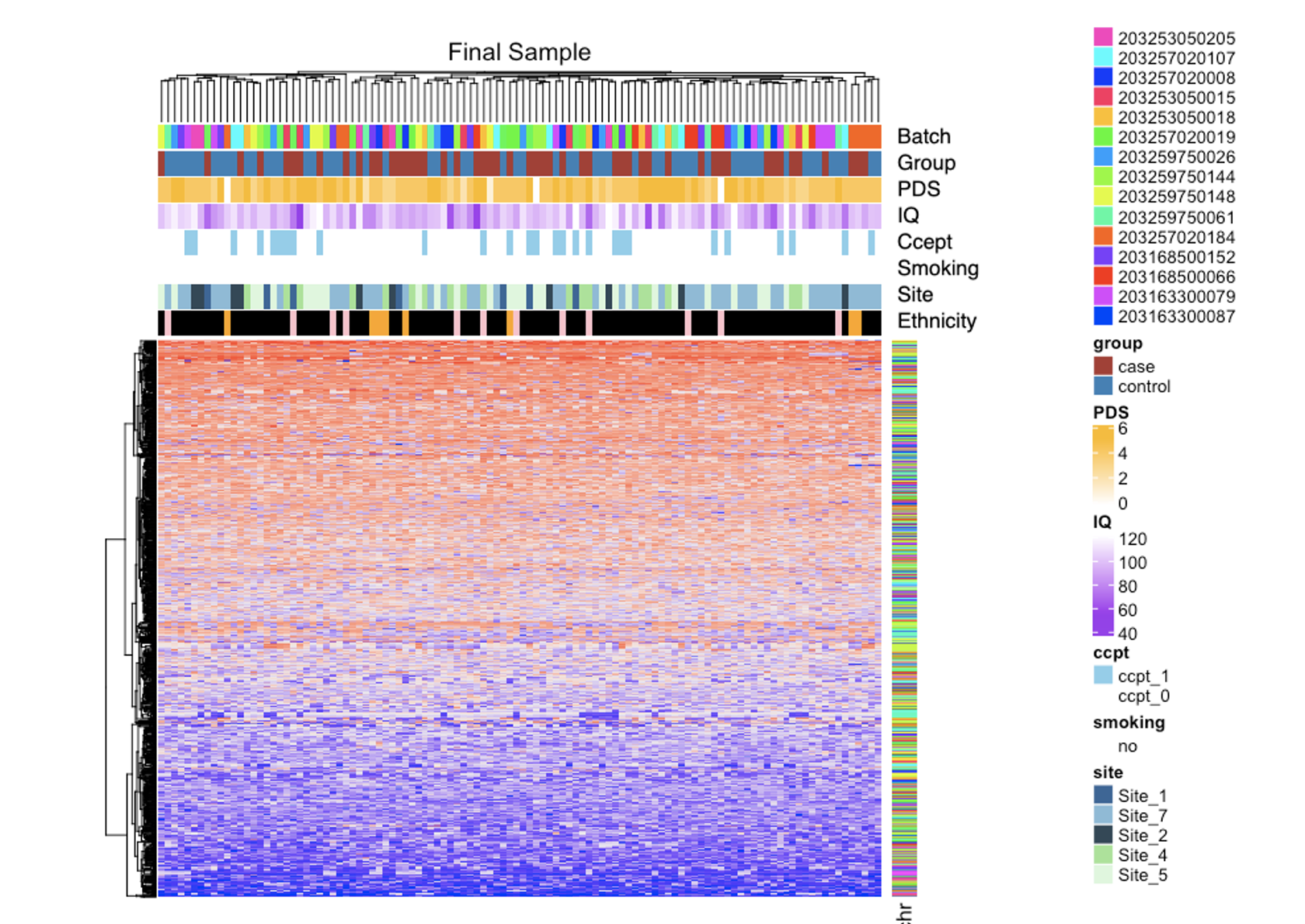


**Supplementary Figure 1 Quality control of final sample**: a) Epigenetic Data post-QC sample correlation check -Heatmap with dendrogram: Visual inspection confirmed neither clustering of methylation values for matched variables, nor batch effects were present in the final sample. b) Histogram plot confirming normally distributed M values for DMR without outliers. c) Hierarchical clustering plot (dendrogram) showing 4 distinct groups in the dataset.

**1b**


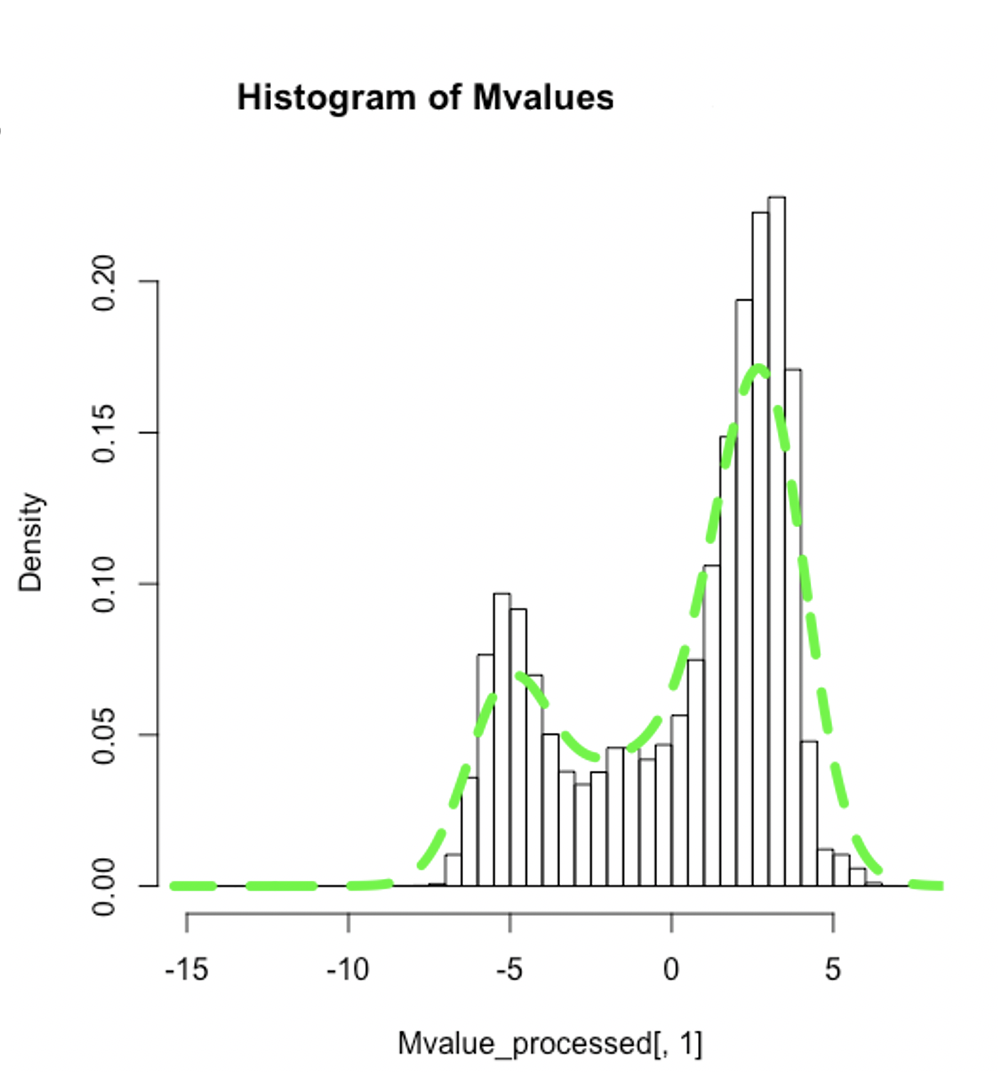


**1c**

**Hierarchical Clustering of DNA Methylation data**


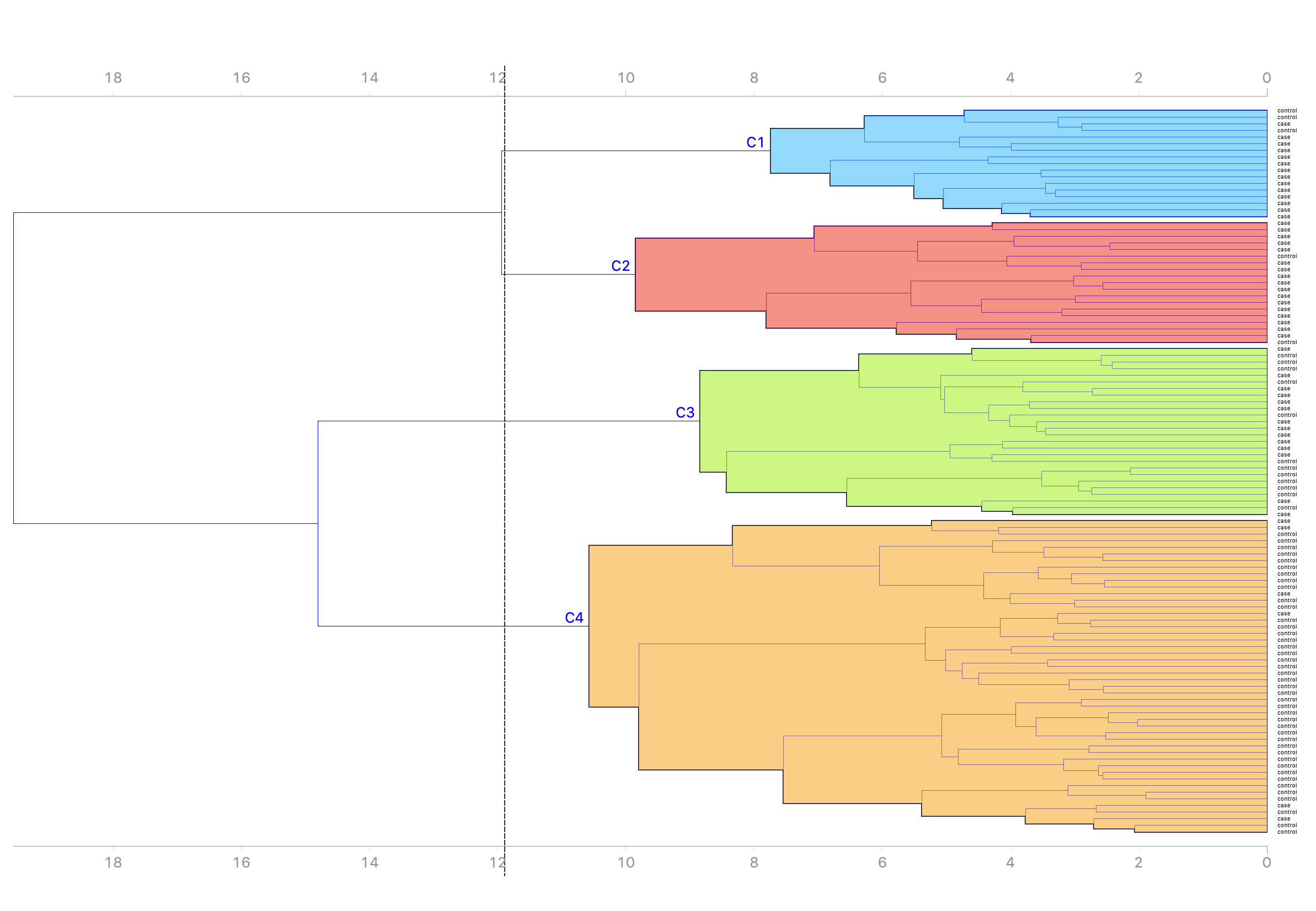


Supplementary Figure 2: QQ plots for 3 EWAS models for (a) Main effect of Group, (b) Main effect of CU, (c) CDxCU interaction

a) **
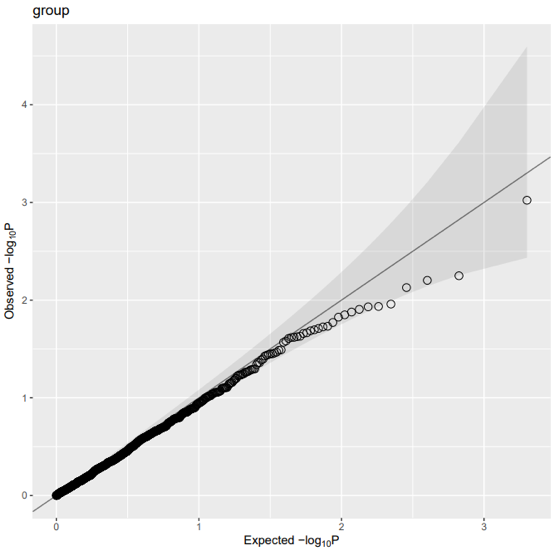
** b)**
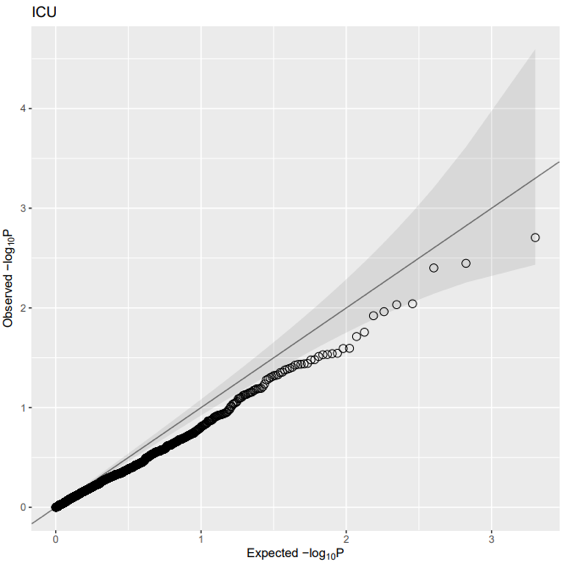
** c)**
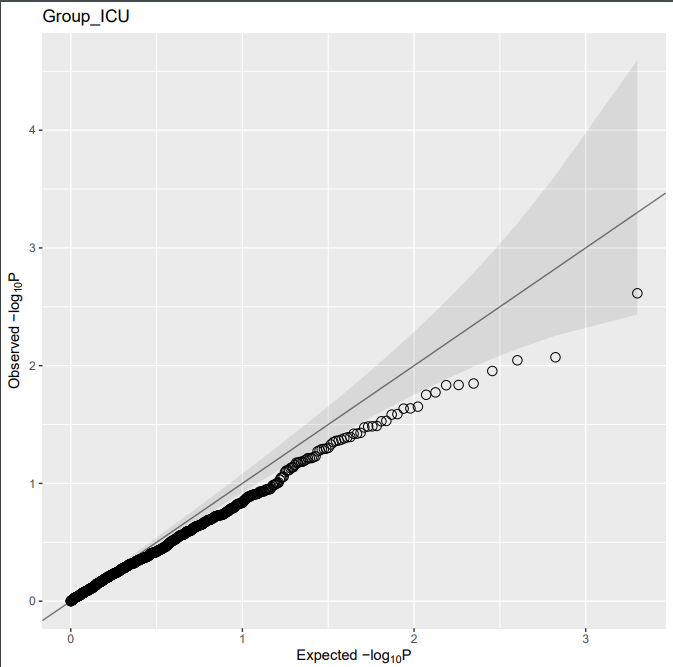
**

**Supplementary Figure 3**: GTEx bar plots of SLC25A24 gene expression across brain tissues.


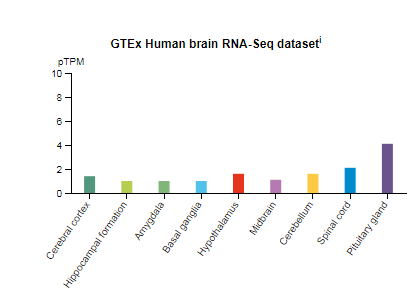


Selection criteria for ROIs for VBM analysis was; (i) region had been previously identified in the literature as of interest in relation to Anti-social behavior/CD /CU traits and, (ii) SLC25A24 gene is reported to be expressed^11^ in post-mortem tissue taken from that brain region

Supplementary Figure 4: Graph to show the relationship between number of CD symptoms and CU score. R^2^= 0.431 across full sample.


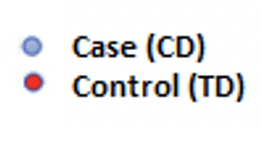


**Supplementary Figure 5:** 3D Rendering of the 4 ROIs tested: the amygdala (green), basal ganglia (yellow), cerebellum (red) and hippocampus (blue)


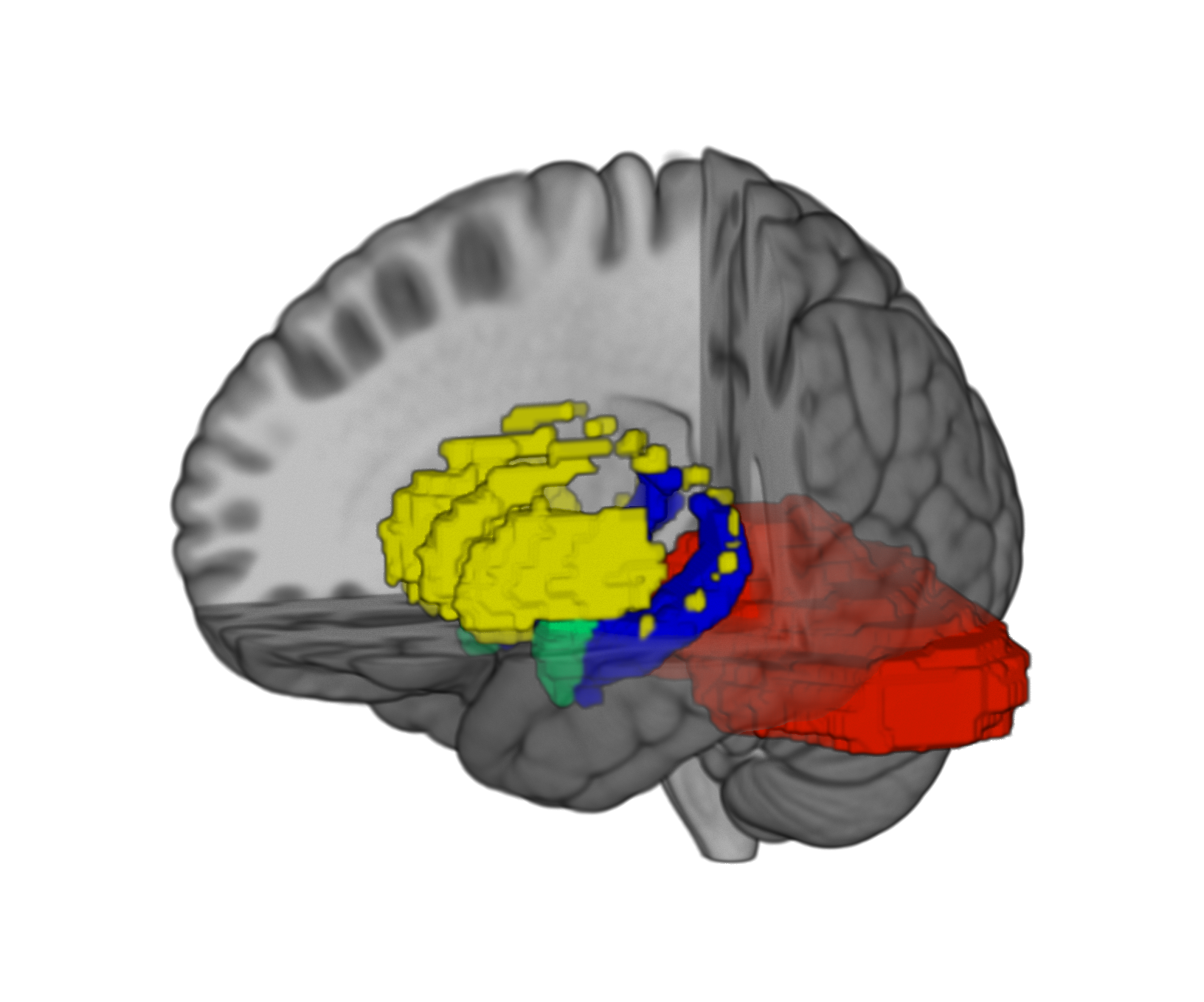

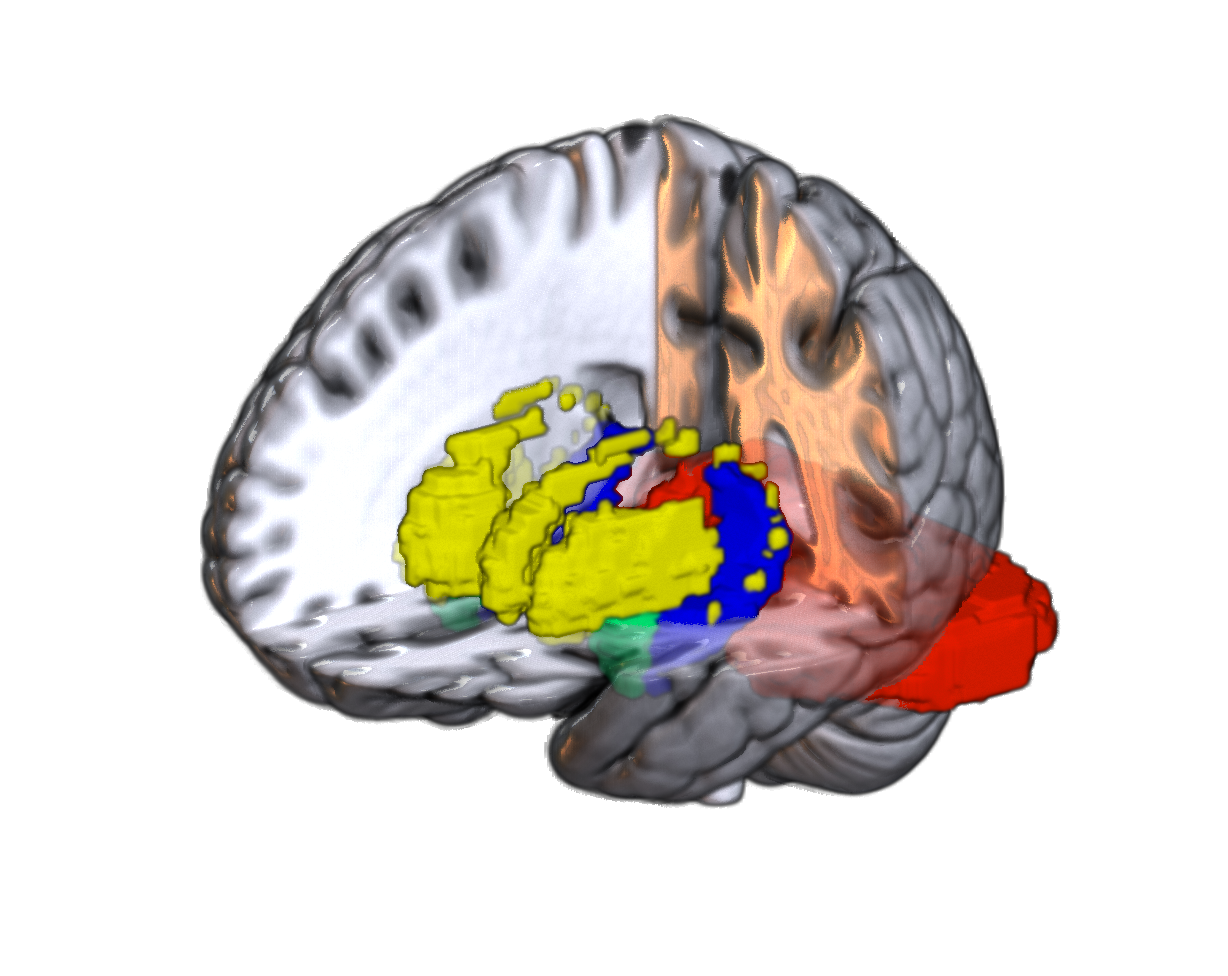

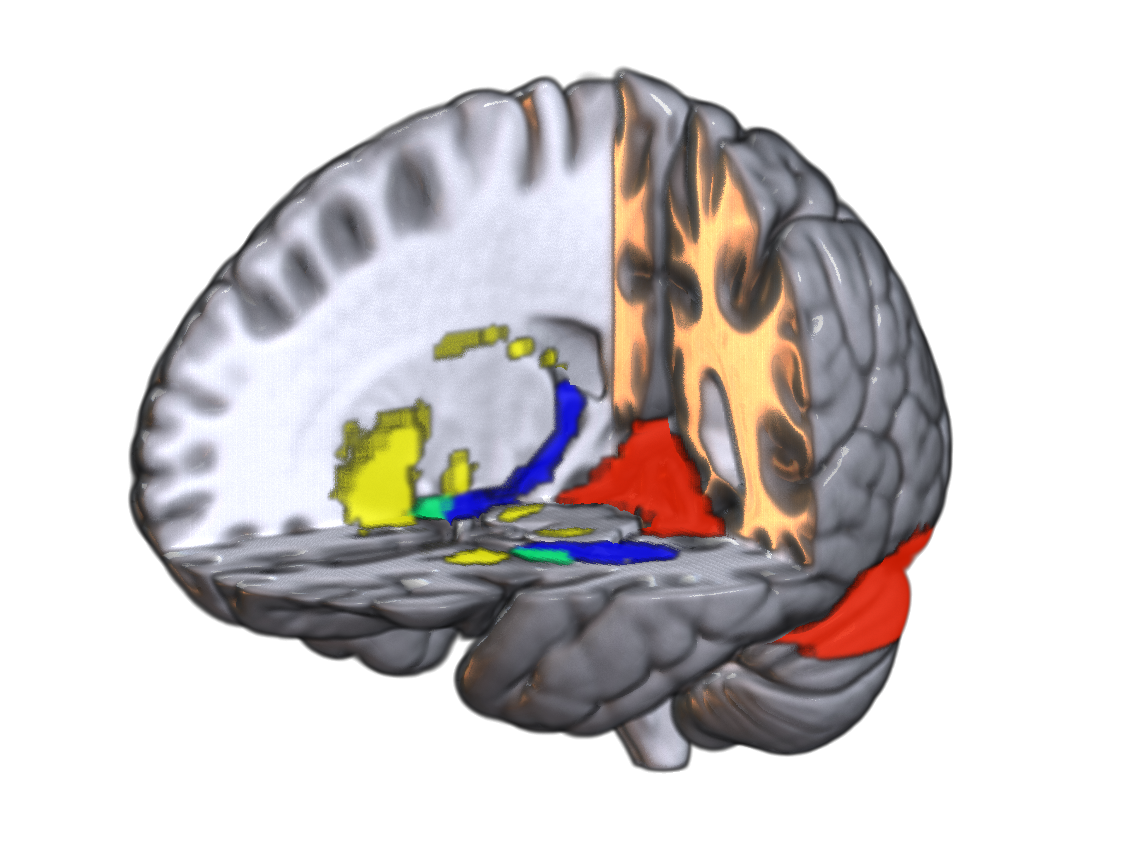


Supplementary References

1 Kaufman, J. *et al.* Schedule for Affective Disorders and Schizophrenia for School-Age Children-Present and Lifetime Version (K-SADS-PL): initial reliability and validity data. *J Am Acad Child Adolesc Psychiatry* **36**, 980-988, doi:10.1097/00004583-199707000-00021 (1997).

2 Wechsler, D. Wechsler Abbreviated Scale of Intelligence–Second Edition. *San Antonio, TX: NCS Pearson* (2011).

3 Wechsler, D. Wechsler intelligence scale for children–Fifth Edition (WISC-V). *San Antonio: Psychological Corporation* (2014).

4 Petersen, A. C., Crockett, L., Richards, M. & Boxer, A. A self-report measure of pubertal status: Reliability, validity, and initial norms. *Journal of youth and adolescence* **17**, 117-133 (1988).

5 Gonzalez-Galarzo, M. C. & Garcia, A. M. [The Spanish version of the International Standard Classification of Occupations-88: discrepancies in the number of rubrics]. *Gac Sanit* **26**, 389, doi:10.1016/j.gaceta.2011.11.011 (2012).

6 Snyman, S., Von Pressentin, K. B. & Clarke, M. International Classification of Functioning, Disability and Health: Catalyst for interprofessional education and collaborative practice. *J Interprof Care* **29**, 313-319, doi:10.3109/13561820.2015.1004041 (2015).

7 Van Casteren, M. & Davis, M. H. Match: a program to assist in matching the conditions of factorial experiments. *Behav Res Methods* **39**, 973-978, doi:10.3758/bf03192992 (2007).

8 Rogers, J. C. *et al.* White Matter Microstructure in Youths With Conduct Disorder: Effects of Sex and Variation in Callous Traits. *J Am Acad Child Adolesc Psychiatry* **58**, 1184-1196, doi:10.1016/j.jaac.2019.02.019 (2019).

9 Wilke, M., Holland, S. K., Altaye, M. & Gaser, C. Template-O-Matic: a toolbox for creating customized pediatric templates. *Neuroimage* **41**, 903-913, doi:10.1016/j.neuroimage.2008.02.056 (2008).

10 Ashburner, J. A fast diffeomorphic image registration algorithm. *Neuroimage* **38**, 95-113, doi:10.1016/j.neuroimage.2007.07.007 (2007).

11 Consortium, G. T. The Genotype-Tissue Expression (GTEx) project. *Nat Genet* **45**, 580-585, doi:10.1038/ng.2653 (2013).

12 Lancaster, J. L. *et al.* Automated Talairach atlas labels for functional brain mapping. *Hum Brain Mapp* **10**, 120-131, doi:10.1002/1097-0193(200007)10:3<120::aid-hbm30>3.0.co;2-8 (2000).

13 Team, R. C. R: A Language and Environment for Statistical Computing - version 3.6.0. (2019).

14 Gentleman, R. C. *et al.* Bioconductor: open software development for computational biology and bioinformatics. *Genome Biol* **5**, R80, doi:10.1186/gb-2004-5-10-r80 (2004).

15 Aryee, M. J. *et al.* Minfi: a flexible and comprehensive Bioconductor package for the analysis of Infinium DNA methylation microarrays. *Bioinformatics* **30**, 1363-1369, doi:10.1093/bioinformatics/btu049 (2014).

16 McCall, M. N. & Almudevar, A. Affymetrix GeneChip microarray preprocessing for multivariate analyses. *Brief Bioinform* **13**, 536-546, doi:10.1093/bib/bbr072 (2012).

17 Fortin, J. P. *et al.* Functional normalization of 450k methylation array data improves replication in large cancer studies. *Genome Biol* **15**, 503, doi:10.1186/s13059-014-0503-2 (2014).

18 Pidsley, R. *et al.* Critical evaluation of the Illumina MethylationEPIC BeadChip microarray for whole-genome DNA methylation profiling. *Genome Biol* **17**, 208, doi:10.1186/s13059-016-1066-1 (2016).

19 Du, P. *et al.* Comparison of Beta-value and M-value methods for quantifying methylation levels by microarray analysis. *BMC Bioinformatics* **11**, 587, doi:10.1186/1471-2105-11-587 (2010).

20 Miller, G. A. & Chapman, J. P. Misunderstanding analysis of covariance. *J Abnorm Psychol* **110**, 40-48, doi:10.1037//0021-843x.110.1.40 (2001).

21 Lupien, S. J., King, S., Meaney, M. J. & McEwen, B. S. Child's stress hormone levels correlate with mother's socioeconomic status and depressive state. *Biol Psychiatry* **48**, 976-980, doi:10.1016/s0006-3223(00)00965-3 (2000).

22 Jaffe, A. E. *et al.* Bump hunting to identify differentially methylated regions in epigenetic epidemiology studies. *Int J Epidemiol* **41**, 200-209, doi:10.1093/ije/dyr238 (2012).
